# Role of plasma neutrophil gelatinase-associated lipocalin as an emerging biomarker of acute renal failure following kidney transplantation and its correlation with plasma creatinine

**DOI:** 10.15171/jrip.2016.21

**Published:** 2016-03-25

**Authors:** Aiyoub Pezeshgi, Sima Abedi Azar, Hussein Ghasemi, Koorosh Kamali, Abdolreza Esmaeilzadeh, Bahare Hajsalimi, Sajad Pour-Asghar, Mohammad Reza Behmanesh, Mina Kiafar

**Affiliations:** ^1^Department of Internal Medicine, Faculty of Medicine, Zanjan University of Medical Sciences, Zanjan, Iran; ^2^Metaolic Diseases Research Center, Zanjan University of Medical Sciences, Iran; ^3^Chronic Kidney Disease Research Center, Tabriz University Of medical Sciences, Tabriz, Iran; ^4^Department of Public Health, School of Public Health, Zanjan University of Medical Sciences, Zanjan, Iran; ^5^Faculty of Medicine, Zanjan University of Medical Sciences, Zanjan, Iran; ^6^Faculty of Medicine, Urmia University of Medical Sciences, Urmia, Iran

**Keywords:** Renal transplantation, Neutrophil gelatinase-associated lipocalin, Acute kidney injury, Rejection

## Abstract

**Introduction:** Graft function early after kidney transplantation is an important parameter in determining the outcome of operation. Urinary and plasma neutrophil gelatinase-associated lipocalin (NGAL), a member of the lipocalin protein family, has been advocated as a sensitive, early biomarker for predicting early renal graft after transplantation. The functions of NGAL appears to be expressed in stress conditions and in tissues undergoing involution. It rapidly accumulates in the kidney tubules and urine after nephrotoxic and ischemic insults.****

**Objectives:** This study aimed to examine the prognostic role of NGAL early after renal transplantation.

**Patients and Methods:** A total of 37 kidney recipients were enrolled from a teaching centre in Tabriz within a 6-month period of time. Plasma NGAL was measured immediately before and at 6 and 12 hours post-transplantation. Changes of serum creatinine were documented daily within the first week post-operation. Acute kidney injury (AKI)/graft rejection during the first week after transplantation was the outcome variable.

**Results:** There were 22 males (59.5%) and 15 females (40.5%) with the mean age of 34.93 ± 14.97 years (range: 12-59) in the study group. AKI/graft rejection developed in 12 patients (32.4%). The mean post-transplantation plasma NGAL levels and serum creatinine at all time points were significantly higher in patients with AKI/graft rejection. The best prognostic role was found for plasma NGAL at 12 hours (sensitivity = 100%, specificity = 92%; cut-off value = 309 ng/ml), far better than the prognostic accuracy of corresponding serum creatinine (sensitivity = 66.7%, specificity = 61.9%).

**Conclusion:** Plasma NGAL, particularly 12 hours after transplantation, is a very sensitive and specific biomarker for predicting acute renal injury.

**Implication for health policy/practice/research/medical education:**

Plasma neutrophil gelatinase-associated lipocalin (NGAL), particularly 12 hours after kidney transplantation, is a very sensitive and specific biomarker for predicting acute renal injury.

## Introduction

Neutrophil gelatinase-associated lipocalin (NGAL) is known as a gene that undergoes rapid changes in the kidneys as a result of ischemic injuries (1,2).

In fact, NGAL is a protein of the lipocalin family which grows through neutrophils and other epithelial cells (including aggregation proximal tubules chains). According to the results of previous research, NGAL is a very useful renal biomarker for the early diagnosis of acute renal failure (ARF) in children and adults undergoing renal implant and cardiac surgery. Based on the existing information, NGAL can be identified in the first urine samples 2 hours after ischemia. Moreover, the NGAL protein is specifically produced in the renal tubule cells and is easily identifiable in plasma and urine of animal samples with acute kidney injury (AKI) (3).

The emergence of NGAL increases drastically in the renal tubules of AKI samples (4,5).

In addition, it was found out that NGAL can be used as a biomarker and an effective predictor for AKI samples in acute clinical conditions (6).

Moreover, results of preliminary studies show that exact assay of urinary NGAL can predict AKI after administration of contrast (7), renal implant (8,9), hemolytic uremic syndrome (HUS) (10), and lupus nephritis (11). It can also predict AKI in patients with severe conditions (12,13) and chronic renal failure (14), and cardiac cases (15). However, recent studies have questioned the sensitivity of urinary NGAL in prediction of ARF after renal implant and have stressed examination of NGAL serum level (16,17).

Development of a standard clinical program for the assessment of NGAL can substantially contribute to renal diagnosis especially in cases in need of intensive care (18,19).

Accordingly, it should be said that the results of studies aimed to determine and approve the role of NGAL as an indicator for the diagnosis of AKI after renal transplantation can cause evolutions in this field. These changes will facilitate the identification of acute renal injury and will provide for a reduction in the incidence of its complication. Moreover, the changes will prevent the development and expansion of the injuries to prevent the incidence of the final phase of renal failure.

## Objectives

While the role of NGAL as a biomarker for predicting acute renal injury has been proved in some studies, considering the possibility of genetic diversity in the NGAL gene in Iran and considering the significance of this phenomenon for the treatment of such patient, this study was necessarily carried out. Findings of this research can provide more insight into the role and effectiveness of the NGAL indicator. This study not only fills the existing research gap but also paves the way for further studies in Iran.

## Patients and Methods

In this study, end-stage renal disease (ESRD) dialysis cases who were candidates for renal implant were studied from 2004 to 2014 in Imam Reza hospital of Tabriz.

A total of 37 patients were selected and studied in this research. In general, the only inclusion criterion for this study was availability of informed consent of participants. The exclusion criteria also included lack of cooperation, early death of the patient before renal performance, and renal implant failure.

After providing the required explanations to the patients the blood samples of patients were obtained before anesthesia and 16 and 12 hours after the surgery. The blood samples were used to determine the plasma NGAL level and serum creatinine level and were sent to the laboratory of the hospital. It is worth mentioning that the serum creatinine level in patients was controlled and examined for at least 7 days.

Considering the persistence of plasma samples at a temperature of -70°C in over one month, in order to increase the precision of the investigation and reduce the number of kits required for calibration of the ELISA device, all of the samples that were sent to the laboratory were centrifuged and the resulting serum sample was stored at a temperature of -70°C after printing codes on the associated microtubes. After completion of sampling all samples were tested simultaneously. All samples were examined in two 3-month periods.

The plasma NGAL level was measured using the Cristal-Day-Biotech kit made in China. The method used for this measurement was the ELISA method (fluorescence de-tected-immunoassay).

The 50% (or higher) increase in the maximum serum creatinine level after operation was considered to be an indicator of AKI as compared to the serum creatinine level before operation or the need for dialysis within 1 week after implant. Accordingly, patients were classified into the groups of patients with AKI and patients without AKI. The plasma NGAL level was measured and compared for the two groups at different time periods.

Research variable included age, gender, underlying causes of renal failure, pre-and post-operative plasma NGAL level (6 and 12 hours after surgery), daily serum creatinine level in the first week, ARF, implant rejection in the first week after implant, and mortality within one week after surgery.

### Ethical issues

The research followed the tenets of the Declaration of Helsinki; all samples were participated after their parent’s satisfaction. Participation in this study was voluntary and patients were thus free to withdraw from the study at any time without having any effect on their treatment process. This study was approved by the ethic committee of Zanjan University of Medical Science. The informed consent of all patients was obtained before including them in the study.

### Statistical analysis

We used descriptive analytical test (Mean± SD), frequency and percentage for presenting descriptive data to compare qualitative data. Chi-square test or Fisher exact test was used. We also evaluated the normal distribution of laboratory data for studying their mean difference and due to their lack of normal distribution we used Mann-Whitney U test. For studying the relationship between plasma NGAL and serum creatinine, Pearson correlation test was used and then we used receiver operating characteristic (ROC) curve for determining the cut off point for NGAL to diagnosing renal failure. In all cases, *P* value less than 0.05 is considered significant.

## Results

Of the patients under study, 22 (59.5%) were male and 15 were female (40.5%). The average age of patients at the time of renal implant was 34.93±14.97 years (12-59 years).

Underlying causes of renal failure included obstruction (12 cases or 32.4%); hypertension (7 cases or 18.9%); diabetes mellitus (4 cases or 10.8%); infection (3 cases or 8.1%); focal segmental glomerulosclerosis (FSGS) (1 case or 2.7%); and polycystic kidney disease (1 case or 2.7%). None specific causes of renal failure was also observed in 9 patients (24.3%).

The average plasma NGAL level before implant, 6 hours after implant, and 12 hours after implant was 311.14±102.69 ng/ml, 317.81±107.28 ng/ml, and 312.16±134.80 ng/ml, respectively.

The plasma NGAL levels measured 6 (*P*=0.70) and 12 (*P*=0.96) hours after kidney transplantation were not significant as compared to the average plasma NGAL level before implant.

The average serum creatinine level that was measured daily during the first week after kidney transplantation was 5.33±2.71 mg/dl, 2.72±1.62 mg/dl, 1.97±1.24 mg/dl, 1.83±1.21 mg/dl, 1.61±0.68 mg/dl, 1.64±1.15 mg/dl and 1.77±1.08 mg/dl on Saturday, Sunday, Monday, Tuesday, Wednesday, Thursday and Friday, respectively. In the course of the study, ARF, kidney transplant rejection and mortality were seen in 2 (5.4%), 7 (18.9%) and 1 (2.7%) patients, respectively.

The correlation of plasma NGAL levels measured 6 and 12 hours after renal implant with serum creatinine level in the first week after surgery is shown in Table 1. In none of the study phases a significant statistical correlation between the variables was observed.

**Table 1:** Correlation between plasma level of NGAL at 6 and 12 hour after transplantation with plasma creatinine during the first postoperative day week

	**NGAL 6 hours**		**NGAL 12 hours**	
	r	***P***	**r**	***P***
1st Day	0.12	0.49	0.22	0.19
2nd Day	-0.05	0.79	0.17	0.32
3rd Day	-0.06	0.79	0.20	0.24
4th Day	-0.07	0.68	0.13	0.45
5th Day	-0.09	0.63	0.17	0.33
7th Day	0.09	0.59	0.18	0.29
8th Day	-0.01	0.94	0.19	0.29

Abbreviation: NGAL, neutrophil gelatinase-associated lipocalin.

In the ARF group, 9 patients (75%) were male and 3 (25%) were female. In the non-ARF group, 13 patients (52%) were male and 12 (48%) were female (*P*=0.29).

The average age of patients with ARF and patients without ARF was 39.90±12.66 and 32.32±15.74 years, respectively (*P*=0.20).

**Figure 1:**
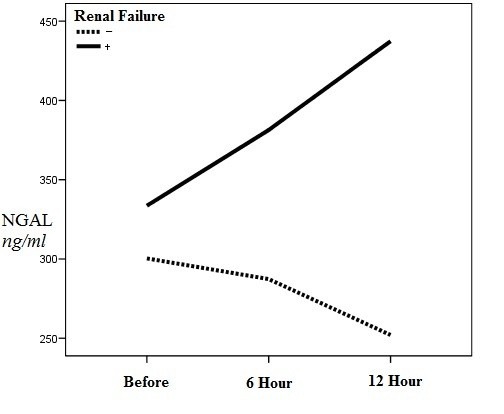
Changes of plasma NGAL at before and after transplantation between two groups with and without renal failure

Average variations of plasma NGAL level before implant and 6 and 12 hours after implant in the participants with and without ARF are shown in Figure 1. According to the results of analysis of repeated measurements, a significant statistical difference between the variations in the two groups was observed (*P*=0.002).

The average plasma NGAL level before transplantation was 333.58±116.30 ng/ml and 300.36±96.15 ng/ml in the ARF and non-ARF groups, respectively (*P*=0.36).

The average plasma NGAL levels 6 hours after implant were 38.33±147.16 ng/ml and 286.32±65.97 ng/ml in the ARF and non-ARF groups, respectively.

The average plasma NGAL level measured 12 hours after implant was 437.33±164.16 ng/ml and 252.08±57.53 ng/ml in the ARF and non-ARF groups, respectively.

The plasma NGAL level measured 6 (*P=*0.01) and 12 (*P*<0.001) hours after transplantation was significantly higher in the ARF group.

**Figure 2:**
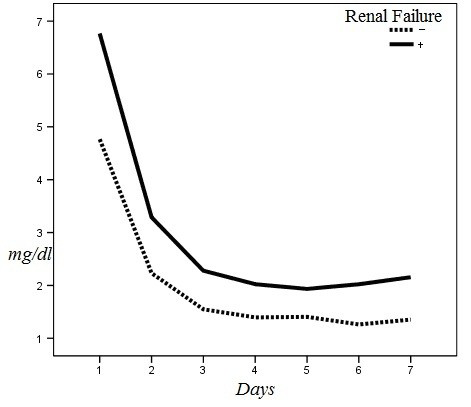
Changes of plasma creatinine at before and after transplantation between two groups with and without renal failure

The changes in serum creatinine level during the first week after implant in the patients with and without ARF are illastrated in Figure 2 (*P=*0.01).

**Figure 3:**
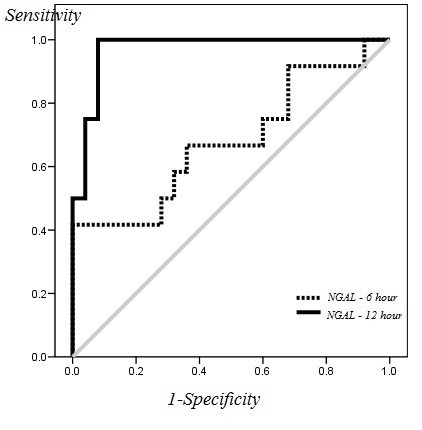
ROC curve of plasma NGAL at after transplantation to predict of ARF.

The ROC curve for plasma NGAL level measured 6 and 12 hours after implant is shown in Figure 3 for predicition of ARF.

The area under the curve for NGAL levels measured 6 and 12 hours after implant was equal to 0.68 (*P=*0.08) and 0.97 (*P*<0.001), respectively. The NGAL level measured 6 hours after implant (cut of point [COP] > 309 ng/ml) had a sensitivity of 66.7% and specificity of 64% in prediction of ARF. The NGAL level measured 12 hours after implant (COP >317 ng/ml) had a sensitivity of 100% and specificity of 92% in prediction of ARF. The ROC curve for serum creatinine level measured during the first week after implant is depicted in Figure 2 for prediction of ARF.

Table 2 presents the area under the curve, sensitivity, specificity and COP of serum creatinine level on a daily basis for one week after the implants.

**Table 2:** Area under the curve and the prediction of creatinine for ARF during the first postoperative day week

	**Area under the curve**	***P***	**Sensitivity (%)**	**Specificity (%)**	**Cut of Point (mg/dl)**
1st Day	0.70	0.09	66.7	61.9	5.2
2nd Day	0.73	0.05	66.7	66.7	2.4
3rd Day	0.76	0.03	66.7	76.1	1.9
4th Day	0.79	0.01	77.8	81	1.7
5th Day	0.77	0.02	88.9	81	1.6
7th Day	0.83	0.004	88.9	81	1.6
8th Day	0.82	0.01	77.8	85.7	1.7

Abbreviation: ARF, acute renal failure.

## Discussion

ARF or delayed graft function is relatively common among the receivers of graft tissues. So far numerous studies have studied the effectiveness of different markers for prediction of this condition.

NGAL is known as a gene that undergoes rapid changes in the kidney following ischemic injuries. In fact, NGAL is a protein of the lipocalin family which grows through neutrophils and other epithelial cells (including aggregation proximal tubules chains). According to the results of previous research, NGAL is a very useful renal biomarker for the early diagnosis of ARF in children and adults undergoing renal implant and cardiac surgery (20,21).

In this research, it was tried to determine the role of plasma NGAL in prediction of ARF during the first week of surgery. According to the findings of this study, the average plasma NGAL level 6 and 12 hours after implant was significantly higher in the ARF group as compared to the group with normal graft tissue performance.

Results of this study comply with the results of other studies on patients receiving renal implants and patients with other clinical conditions such as patients undergoing cardiac surgery (22) or severely sick patients kept in intensive care unit (ICU) (23).

In the study by Kusaka et al (24) the serum NGAL level helped predict the failure of the graft tissue in a short white after renal implant. Accordingly, in the study by Lebkowska et al (20) the precision of serum NGAL in prediction of post-transplant ARF was studied in 41 patients. The serum NGAL level before and after renal transplant was measured in days 1, 3, 6 and 10.

In this research, a significant decline was observed in serum NGAL level on the first day after implant. In all time periods a significant correlation between serum NGAL and creatinine levels was observed. However, the serum level of NGAL did not decline considerably in patients with delayed graft function. Accordingly, it is recommended to use this serum variable especially as an early indicator (predictor) for prediction of renal graft function.

It is worth mentioning that, unlike the results of the above study, no significant correlation between plasma NGAL level and serum creatinine levels after renal transplantation was observed. This can be ascribed to the early diagnostic capability of NGAL as compared to creatinine.

Malyszko et al (25) studied the relationship between serum creatinine level and estimated glomerular filtration rate in 90 patients who received renal implant. They stated that serum NGAL can be used as an early sensitive marker for prediction of renal graft function.

In the study by Mahdavi-Mazdeh et al (16), serum NGAL level was measured in 52 patients with renal transplant in 0, 2, 4, 12, and 18 hours after transplantation. They detected that serum NGAL level is significantly correlated to serum creatinine level especially 2 hours after implantation. Hence, this parameter can be used as a predictor for prediction of renal graft function.

In another study by Mahdavi-Mazdeh et al (26) the serum NGAL and creatinine levels were measured to predict post-graft renal recovery. It was found out that the serum creatinine and NGAL levels were higher in patients with delayed graft function (6 patients) and slow graft function (9 patients) as compared to patients with immediate graft function. However, the difference was only statistically significant in the case of NGAL. Results of the ROC-based investigations showed that the highest under the curve area belonged to NGAL measured 24 hours after the implantation (0.82).

In the present study, the diagnostic power of serum NGAL level was measured using ROC curves. The area under the curve obtained for NGAL was 0.68 and 0.97 in 6 and 12 hours after the implant, respectively.

As seen, based on the results of the above study, the area under the curve for NGAL in this study is larger than that of NGAL obtained 24 hours after implant. However, it is worth noting that the two studies employed two different target variables and the difference can perhaps explain the discrepancies in results. Moreover, in the present study the variables were only measured 6 and 12 hours after operation which can be considered among the limitations of this research. However, it shall be mentioned that the sensitivity and specificity calculated for NGAL 12 hours after operation for prediction of ARF were 100% and 92%, respectively (COP=217 ng/ml). This result is an extraordinary finding for selecting the factor predicting prognosis.

In a meta-analysis by Haase et al (13) through the results of 19 studies on the role of serum NGAL in prediction of renal graft function, they found NGAL can properly predict ARF. Moreover, it was stated that the diagnosis precision obtained with serum NGAL in the investigations was 77.5% in 24 to 48 hours before the incidence of ARF. The corresponding COP also varied between 100 and 270 ng/ml.

Poorshahbaz et al (27) found that the frequencies of patients prone to AKI at the first, third, fifth and seventh day of administration of gentamicin were 0%, 8.1%, 18.9%, and 13.5%, respectively. No statistically significant difference was observed between NGAL levels before and after the administration of gentamycin (*P*=0.082).

As seen, our findings in this regard comply with the results of other studies.

The prognostic power of NGAL was considerably higher than that of simultaneous serum creatinine (on the first day after implantation).

As mentioned, this finding highlights the significance of early measurement of plasma NGAL as a sensitive indicator as compared to creatinine.

## Conclusion

In summary, it can be concluded that results of the present study showed that by measuring serum NGAL level with considerable specificity and full sensitivity it is possible to predict incidence of ARF after renal graft. Therefore, this indicator can be used in clinical examinations.

## Limitations of the study

It shall be mentioned that although prior to this study the required sample size was estimated based on the results of the strength test, further studies with larger samples are needed to obtain the ideal COP.

Moreover, although it is appropriate to measure serum NGAL level 12 hours after implantation based on the resulting diagnostic sensitivity and specificity, it is recommended to increase time periods for measurement of serum NGAL level in future research.

## Acknowledgements

The authors wish to thank Chronic Kidney Disease Research Center, Tabriz University of Medical Sciences, and the Metabolic Diseases Research Center, Zanjan University of Medical Sciences, to support this study and all the colleagues and nurses of Kidney Transplantation Ward of Emam Reza hospital, Tabriz, Iran who participated in the data collecting process

## Authors’ contribution

All authors contributed equally to the paper.

## Conflicts of interest

The authors declare that they have no conflicting interest.

## Ethical considerations

Ethical issues (including plagiarism, data fabrication, double publication) have been completely observed by the authors.

## Funding/Support

This manuscript supported financially by Metabolic Disease Research Center, Zanjan University of Medical Sciences, Iran.
